# The Plant Hormone Cytokinin Confers Protection against Oxidative Stress in Mammalian Cells

**DOI:** 10.1371/journal.pone.0168386

**Published:** 2016-12-22

**Authors:** Eman M. Othman, Muhammed Naseem, Eman Awad, Thomas Dandekar, Helga Stopper

**Affiliations:** 1 Institute of Pharmacology and Toxicology, University of Würzburg, Würzburg, Germany; 2 Department of Analytical Chemistry, Faculty of Pharmacy, University of El-Minia, Minia, Egypt; 3 Department of Bioinformatics, Biocenter, University of Würzburg, Am Hubland, Würzburg, Germany; 4 Bogazici University, Department of Molecular Biology and Genetics, Kuzey Park, Istanbul; University of PECS Medical School, HUNGARY

## Abstract

Modulating key dynamics of plant growth and development, the effects of the plant hormone cytokinin on animal cells gained much attention recently. Most previous studies on cytokinin effects on mammalian cells have been conducted with elevated cytokinin concentration (in the μM range). However, to examine physiologically relevant dose effects of cytokinins on animal cells, we systematically analyzed the impact of kinetin in cultured cells at low and high concentrations (1nM-10μM) and examined cytotoxic and genotoxic conditions. We furthermore measured the intrinsic antioxidant activity of kinetin in a cell-free system using the Ferric Reducing Antioxidant Power assay and in cells using the dihydroethidium staining method. Monitoring viability, we looked at kinetin effects in mammalian cells such as HL60 cells, HaCaT human keratinocyte cells, NRK rat epithelial kidney cells and human peripheral lymphocytes. Kinetin manifests no antioxidant activity in the cell free system and high doses of kinetin (500 nM and higher) reduce cell viability and mediate DNA damage *in vitro*. In contrast, low doses (concentrations up to 100 nM) of kinetin confer protection in cells against oxidative stress. Moreover, our results show that pretreatment of the cells with kinetin significantly reduces 4-nitroquinoline 1-oxide mediated reactive oxygen species production. Also, pretreatment with kinetin retains cellular GSH levels when they are also treated with the GSH-depleting agent patulin. Our results explicitly show that low kinetin doses reduce apoptosis and protect cells from oxidative stress mediated cell death. Future studies on the interaction between cytokinins and human cellular pathway targets will be intriguing.

## Introduction

Cytokinins (CKs) are an adenine as well as non-adenine derived heterogeneous class of regulatory molecules that participate in almost every aspect of plant biology. The CK signal is perceived by membrane-located sensor histidine kinases and transmitted through a two-component system (TCS) which is among the higher eukaryotes unique to plants [1, 2]. Besides plants, many plant-interacting microbes, such as various types of pathogenic and non-pathogenic bacteria, some fungi, nematodes and animal pathogens such as *Mycobacterium tuberculosis* have been shown to produce CK [3, 4]. In spite of their bright prospects for applied research, the presence of CKs, their signaling circuitry and their biological functions in mammalian cells are largely uncharacterized. Kinetin is used in our study as a synthetic CK but was identified also in the endosperm liquid of fresh young coconut fruits, in plant cell extracts and human urine and some other biological extracts. It has been extensively used as growth promoting hormone in plant tissue culture systems [1, 2] but the potential effects of kinetin on human health are still under research. Some studies reported an effect of kinetin on human disorders or diseases [5–8]. Examples are prevention of age-related changes in human skin, presumably by protecting the DNA in skin cells from damage and by decreasing skin water loss [5, 8] as well as the therapeutic potential of kinetin in the treatment of the human splicing disease familial dysautonomia, a genetic disorder affecting the nervous system [6, 9]. It has also been reported that kinetin influences oxidative stress parameters in human fibroblasts *in vitro* [10]. Despite these various fragmentary reports on the application of CKs, their physiological effects on mammalian cells have not been congruently analyzed. We systematically assess the protective effects of kinetin in mammalian cells in a dose dependent manner; pointing out toxic and DNA damaging effects for higher concentrations as well as their protective effects for low concentrations.

Oxidative stress is a state of imbalance between oxidant production and antioxidant defense capacity of a cell. It has been described as a risk factor in a number of human diseases such as atherosclerosis, cancer, neurodegenerative diseases, and aging [11, 12] and in various pathogenic processes [13, 14]. Reactive oxygen species (ROS) in cells can attack the DNA leading to oxidative DNA damage which may result in mutation. Furthermore, ROS affect cellular protein and lipid molecules, compromising their function and leading to the production of cytotoxic lipid peroxidation products.

Antioxidants have been used to protect cells against ROS, and natural antioxidants have been investigated for their use as preventive and therapeutic agents in medicine [15–17].

In this context, we characterized the intrinsic properties as well as different protective activities of kinetin in mammalian cells such as HL60 cells, HaCaT human keratinocyte cells, NRK rat epithelial kidney cells and human peripheral lymphocytes.

Furthermore, we measured the intrinsic antioxidant activity of kinetin in a cell free system using the FRAP assay and in cells using dihydroethidium (DHE) staining, and its ability to act as antioxidant against NQO that mediates oxidative stress.

The ability of kinetin to induce DNA damage by itself was also examined by using the comet assay, followed by assessment of its antigenotoxic activity against oxidative stress that mediates DNA damage. We delineate the potential for CKs binding proteins in mammalian sera as important cellular targets for CK mediated protection against oxidative stresses in future experiments. The detailed assessment of the complex kinetin effects provides a solid foundation for further studies in human cells.

## Materials and Methods

### Chemicals

The chemicals kinetin and sodium arsenite were obtained from Sigma-Aldrich (Dorset, Germany). 4-Nitroquinoline 1-oxide (NQO-sc256815) was purchased from Santa Cruz Biotechnology (Heidelberg, Germany), dihydroethidium (DHE) was purchased from Merck Biosciences GmbH (Schwalbach, Germany). Gel Red and Gel Green were purchased from Biotrend (Köln, Germany). Annexin V was purchased from Roche (Mannheim, Germany). Cell culture media (RPMI- 1640) and reagents were obtained from PAA Laboratories GmbH (Pasching, Austria) and Invitrogen Life Technologies (Carlsbad, California, and Darmstadt, Germany).

### Cell culture and primary cell preparation

HL-60, a human promyelocytic cell line [18], was kindly donated by Prof Schinzel (Vasopharm GmbH). HL-60 cells were grown in 5% (vol/vol) CO2 in RPMI 1640 medium at 37°C, supplemented with 10% (vol/vol) fetal bovine serum, 1% (wt/vol) L-glutamine, and 0.4% (wt/vol) antibiotics (50-U/mL penicillin and 50-mg/mL streptomycin). The cells were routinely subcultured twice per week.

HaCaT, human keratinocytes cell line [19] were obtained from the Cell line service (Eppelheim, Germany). HaCaT cells were cultured at 37°C, 5% (v/v) CO_2_ in DMEM high glucose (4.5 g/L) supplemented with 10% (v/v) fetal bovine serum (FBS), 1% (w/v) L-glutamine, and 0.4% (w/v) antibiotics (50 U/ml penicillin, 50 mg/ml streptomycin). They were subcultured two times per week.

NRK, an epithelial rat kidney cell line [20], was obtained from the American Type Culture Collection (ECACC,Salisbury, UK) and cultured at 37°C, 5% (v/v) CO_2_ in DMEM high glucose (4.5 g/l) with 10.0% (v/v) FCS, 1.0% (v/v) L-glutamine, 1.0% (w/v) non-essential amino acids and 1.0% (v/v) antibiotics (50 U/ml penicillin, 50 mg/ml streptomycin). They were subcultured twice per week.

### Lymphocyte isolation

Lymphocyte isolation was performed by adding 7 mL of blood from healthy human volunteers, 1:1 as a layer over Histopaque-1077. Centrifugation (400 x g) at room temperature for 30 min, separated the layers, from which mononuclear cells were collected from the plasma/ Histopaque interface. After washing twice with lymphocyte medium (250 x g; 1300 rpm; room temperature for 10 min) they were resuspended in 3 ml of lymphocyte medium. Lymphocytes were treated for 24 hr with 100 nM kinetin and for 30 min with 0.6 μM NQO and comet assay was performed.

### Vitality test

The vitality assay was performed as previously described with minor modification [21]. Briefly, ethidium bromide was replaced with Gel Red (Biotrend, Köln, Germany). Vitality staining was performed for the cells treated with different concentrations of kinetin (1, 3.2,10,32,100, 500, 1000 and 10000 nM) for 24 hr. 3.5 × 10^5^ cells were seeded in 6-well plates for 24 hr in a control medium. After treatment, cells were collected, and 70 μL of the cell suspension was stained with 30 μL staining solution. 20 μl of this mixture was applied to the slide, and the fractions of green and red cells in a total of 200 cells were counted at a 500-fold magnification with a fluorescence microscope.

### Ferric reducing antioxidant power (FRAP) Assay

In the FRAP assay, the reducing capacity of the tested compounds to reduce Fe^3+^ to Fe^2+^ was measured, which is considered to be an important parameter for antioxidant function. This method has been widely used for a rapid assessment of the antioxidant potential of various compounds, beverages and natural products [22]

Total antioxidant activity was measured according to [23]. In brief, the FRAP solution was freshly prepared. Different concentrations of kinetin (1, 3.2,10,32,100,500, 1000 and 10000 nM) were mixed with 600 μL of the FRAP solution and the volume was completed to 800 μl with water. Absorbance was measured at 595 nm after 6 min of incubation at room temperature by a spectrophotometer (Bio-Tek, Model Uvikon XL) against a blank of distilled water. 50 μM Tempol was used as a positive control. FRAP values were obtained by comparing the absorbance change at 595 nm in test reaction mixtures with those containing ferrous ions in known concentrations.

### GSH measurement

GSH content of cells was measured by flow cytometry. 5 × 10^5^ cells were seeded the day before and treated for the indicated times with 100 nM kinetin and for 4 hr with 5 μM patulin, washed in PBS and incubated with 300 μL 400 μM monochlorobimane (MCB) solution in PBS for 30 min on ice. Afterwards cells were washed twice, resuspended in PBS and analyzed by flow cytometry using a LSR I (Becton-Dickinson, Mountain View, CA, USA). Fluorescence intensities of 20,000 cells were recorded. The shift to the right of the fluorescent histograms indicates an increase of cellular GSH content. Mean intensities of peaks were used for statistical analysis.

### Comet assay

The comet assay was carried out as described by Singh *et al*. [20] with slight modifications using a fluorescence microscope (Labophot 2; Nikon GmbH, Düsseldorf, Germany) at 200-fold magnification using image analysis software (Komet 5; BFi OPTiLAS, Gröbenzell, Germany). Briefly, cells were treated according to the experimental design with (i) different concentrations (1, 3.2, 10, 32,100 and 500 nM) of kinetin for 24 hr. (ii) 100 nM kinetin for 24 hr, 0.6 μM NQO or 50 μM H_2_O_2_ for 30 min, and combination of kinetin and the reagents. After the cells were harvested, 20 μL of the treated cell suspension were mixed with 180 μL of 0.5% low-melting agarose and added to fully frosted slides that had been covered with a bottom layer of 1% normal melting point agarose. The slides were incubated in lysis solution (2.5M NaCl, 0.1M EDTA, 0.01M Tris, and 10-g/L N-lauroylsarcosine sodium salt adjusted to pH 10 with NaOH) with 1% Triton X-100 and 10% dimethyl sulfoxide at 4°C. After at least 1 hr, the slides were washed and then placed in the electrophoresis solution (300 mM NaOH and 1 mM EDTA [pH 13.0]) for 20 min. Then the electrophoresis was conducted for 20 min at 25 V (1.1 V/cm) and 300 mA. The slides were neutralized in 0.4M Tris buffer (pH 7.5) and then dehydrated in cold methanol for 10 min at -20°C. The slides were dried at37°C in an incubator and then stored at room temperature.

After DNA staining of each slide with Gel Red/diazabicyclo octane (DABCO) solution (20 μg/mL), images of 100 randomly selected cells (50 per replicate slide) for each sample were analyzed, % tail DNA being the evaluation parameter.

### Microscopic analysis of ROS production

Evaluation of the formation of ROS was performed using the cell-permeable fluorogenic probe DHE. One day before the experiment, 3 x 10^5^ cells were seeded in 6-well plates in 3 mL medium; after treatment of the cells with 100 nM kinetin for 24 hr, the cells were incubated in the dark at 37°C with 0,6 μM NQO, the combination and 10 μM DHE for 30 min. After that, ROS production was detected using a Neubauer chamber under an Eclipse 55i microscope (Nikon GmbH) and a Fluoro Pro MP 5000 camera (Intas Science Imaging Instruments GmbH, Göttingen, Germany) at 200-fold magnification. All DHE staining images were taken using the same exposure time. Quantification was carried out by measuring gray values of 200 cells per treatment using ImageJ 1.40g (http://rsb.info.nih.gov/ij/).

### Detection of apoptosis

#### Microscopic analysis of apoptosis

HL-60 cells were treated for 24 hr with 100 nM kinetin, for 8 hr with 10 μM Na-arsenit and the combination of both. For assessment of apoptosis frequencies, all cells were collected (without washing steps, to avoid losing apoptotic cells), brought onto slides by cytospin centrifugation and fixed by incubation in methanol at -20°C for at least 2 hr. Cells were stained in the dark for 7 min with a gel green (1:100) solution, slides were washed with PBS, and mounted with diazabicyclo-octan (DABCO) for microscopy. Apoptotic cells were examined morphologically in 1,000 cells per slide. For each experiment, two slides were analysed. The average was calculated within three independent experiments.

#### Apoptosis detection by Annexin V

1 x 10^6^ cells were cultured one day before the experiment. On the day of the experiment; cells were treated with 100 nM kinetin for 24 hr and with 10 μM Na-arsenite for 8 hr and with the combination of both substances. At the end of the incubation time the cells were washed with the 1x binding buffer (10x binding buffer: 0,1 M Hepes/NaOH, 140 mM NaCl, 25 mM CaCl_2_, pH 7,4, sterilized) followed by washing with PBS. 100 μl AnnexinV/PI (propidium iodide) suspension (20 μl Annexin V-Fluos Stock, 20 μl PI stock (50 μg/ml), 960 μl 1x binding buffer) was added for 20 minutes at room temperature in the dark. After staining, 900 μl 1x binding buffer were added and 20,000 cells were examined by flow cytometry using a FACS LSR I (Becton-Dickinson, FACScan, USA).

## Statistics

Data are from 3 coded independent experiments and are shown as averages ± standard deviation. The mean of % tail DNA calculated from all 100 cells per treatment was used in the comet assays. Relative values are shown, the average of all control values was set to 1.0, and then all individual experimental values calculated as alterations (x-fold) compared to that. Statistical significance among multiple groups was tested with Kruskal-Wallis test. Individual groups were then tested using the Mann Whitney U-test and results were considered significant if the *p*-value was ≤ 0.05.

## Results and Discussion

Pharmacological activities of natural compounds are of strong interest. A group of small molecule compounds that has not been investigated thoroughly yet are the plant growth regulating hormones. Kinetin, as a model compound of the class CKs, was investigated here concerning its protective activity in physiologically relevant concentrations in cultured human cells.

**Fig 1:**
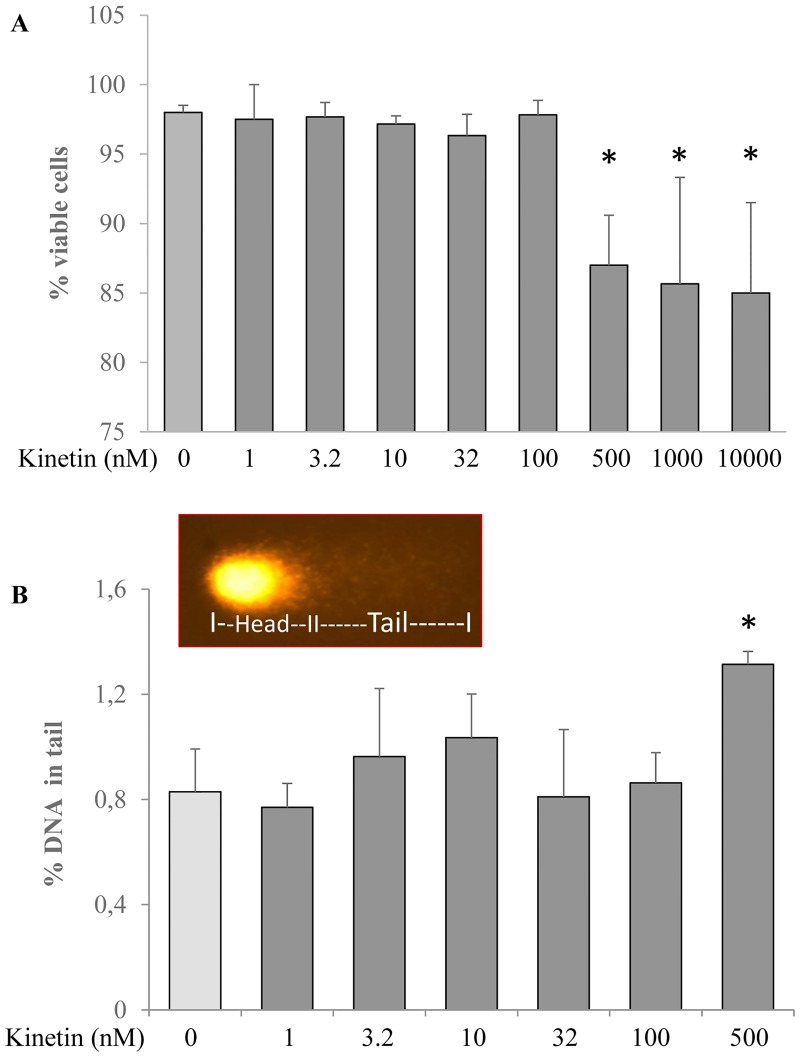
**A)** Vitality test for HL-60 cells treated for 24 hr with different concentrations of kinetin (1, 3.2, 10, 32, 100, 500, 1000 and 10000 nM). B) DNA damage (% DNA in tail) measured by comet assay in HL-60 cells treated with different concentrations of kinetin (1–500 nM) for 24 hr, representative pictures for the comet assay were inserted in the figure.* = significantly different from control.

As a first step in the use of mammalian cultured cells for experiments with kinetin, a vitality test was performed. HL-60 cells were treated with different concentrations of kinetin (1–10000 nM) for 24 hr and percentages of viable and dead cells were quantified. Cells did not suffer from significant toxicity after 24 hr treatment with most of the examined concentrations of kinetin except for 500 nM and higher (Fig 1A). The treatment duration was extended to 48, 72 and 96 hr with 100 nM kinetin, and again no significant effect was observed on cell viability in comparison to the control. Our results are in line with what was reported by Mehrzad et. al [24] who showed that kinetin in micromolar concentration is cytotoxic to MCF-7 cells after treatment for 96 hr and by Griffaut et al [25] who studied the cytotoxicity of kinetin and kinetin riboside, again in micromolar concentration on mouse, human and plant tumors. To generalize the effect CKs have on mammalian cells and to further confirm the effect of the high dose of the kinetin on normal, non-transformed mammalian cells, cell viability was measured in HaCaT and NRK cells after the treatment for 24 hr with 100 nM, 500 nM, 1 μM and 10 μM kinetin. The treatment resulted in significant reduction of viability (6.0 to 8.0% in HaCaT cells and 5.0 to 7.0% in NRK) in comparison to the control upon treatment with 500 nM, 1 μM and 10 μM, whereas 100 nM kinetin did not affect the cell viability (Fig 1A).

As a next step, the ability of kinetin to induce DNA damage was examined by treating HL-60 cells with different concentrations of kinetin (1–500 nM) and the induced DNA damage was measured by comet assay analysis (Fig 1B). Again, 500 nM kinetin mediated significant DNA damage in comparison to the control while the other concentrations did not induce DNA damage in the cultured cells. A published finding [26] is that 10 μM kinetin riboside induced genotoxic stress in mammalian cells, supporting our results of kinetin causing DNA damage at high concentrations. It is well known that many antioxidants can exert toxic effects at supra-physiological concentrations [27]. Therefore, the concentration of 500 nM was excluded from further experiments in which 100 nM kinetin was used to investigate its possible protective acitivites in various cell lines.

Oxidative stress is one of the most popular risk factors in developing different diseases [11] and the antioxidants are among the most non-prescribed drugs sold nowadays. Antioxidants can attenuate oxidative stress and also at high concentrations stimulate ROS production and induce oxidative stress which could be harmful to the cells [28]. The intrinsic antioxidant capacity of kinetin was first assessed in a cell free system using the ferric reducing antioxidant power (FRAP) assay. While the positive control with 50 μM tempol yielded a value of 0.5 (with control set to zero), kinetin at concentrations between 1 nM and 500 nM caused no significant increase in absorption over control. Our results indicated that kinetin exhibits no intrinsic antioxidant (Fe^3+^-reducing) activity over a wide range of concentrations in a cell free system as confirmed by our FRAP assays (no activity detected)).

**Fig 2:**
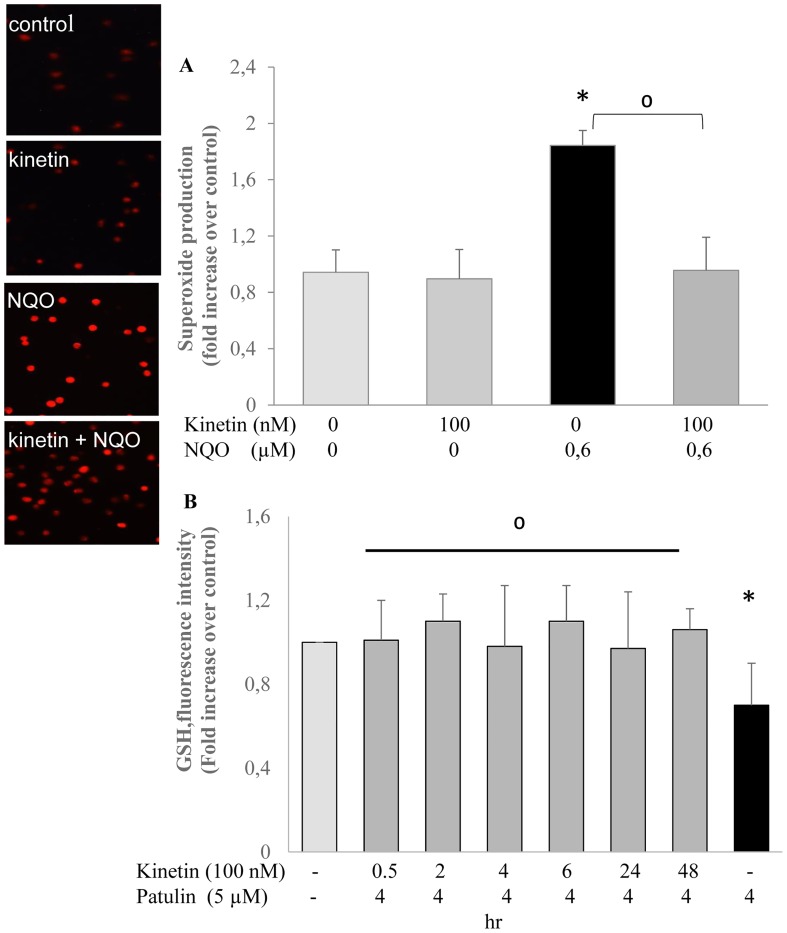
A) Left: microscopic detection of superoxide formation using the dye DHE in HL-60 cells treated for 24 hr with 100 nM kinetin, 0.6 μM NQO and the combination of both and 30 min 10 μM DHE; right: the pictures represent the stained cells; B) Cellular GSH level after 24 hr incubation with 100 nM kinetin and for 4 hr with 5 μM patulin. Analysis was done by flow cytometry using the dye monochlorobimane. * = significantly different from control, ^0^ = significantly different from the NQO or patulin.

In the cellular assays, two end points were detected to examine the antioxidant activity of kinetin. First, ROS production was stimulated in the cells by using NQO (4-nitroquinoline 1-oxide), and was detected by DHE (dihydroethidium) staining. Upon treatment of the cells with NQO, stimulation of ROS production and oxidative stress was observed as higher red fluorescence in comparison to control or kinetin alone. Pre-treatment of the cells with kinetin attenuated the NQO mediated ROS production and protected the cells from the oxidative stress (Fig 2A). Second, GSH level was determined by flow cytometric analysis. The mycotoxin patulin was used as the positive control, and treatment of the cells with patulin for 4 hr reduced the level of the cellular GSH while the pretreatment of the cells with kinetin protected the cells against this effect (Fig 2B). The protection from depletion of GSH by kinetin may indicate a possible mechanism for the antioxidative activity of kinetin and our observations are in line with the findings of Jabłońska-Trypuć, et al. (2016), who reported that kinetin influenced the GSH level in untreated human skin fibroblasts [10].

**Fig 3:**
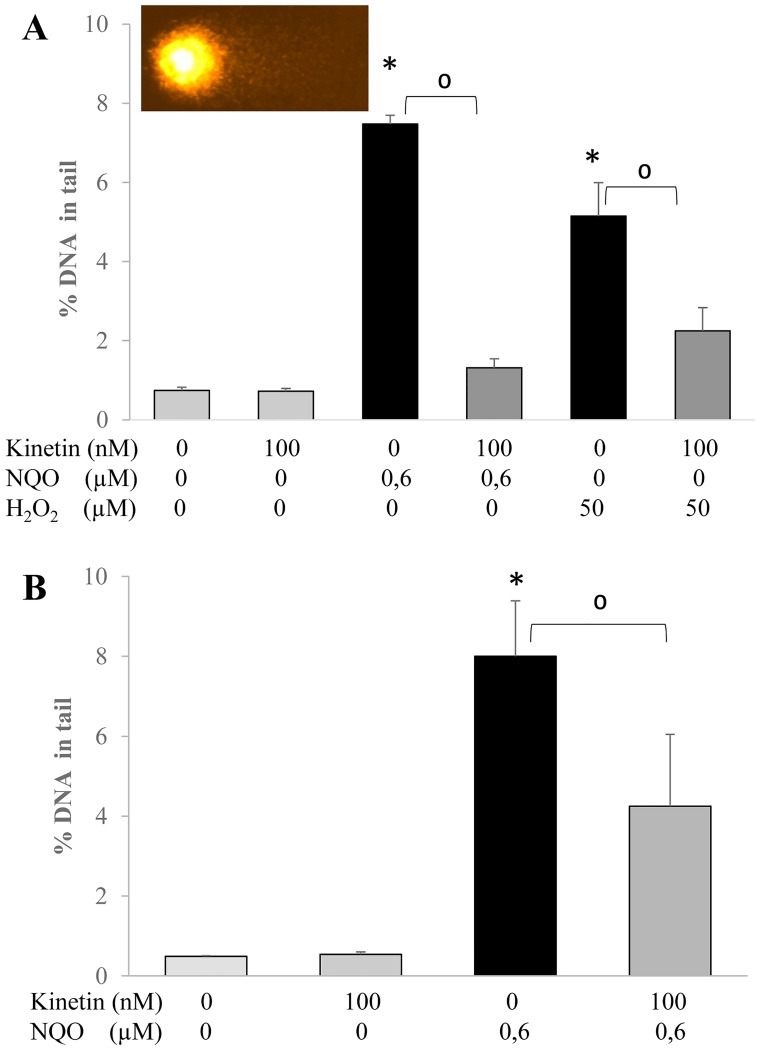
DNA damage (% DNA in tail) measured by comet assay in HL-60 cells treated with A) 100 nM kinetin for 24 hr, 0.6 μM NQO, 50 μM H_2_O_2_ for 30 min, and combination of kinetin and the reagents without washing step between kinetin and the three reagents, B) 100 nM kinetin, 0.6 μM NQO and the combination of both with washing step in-between kinetin and NQO. * = significantly different from control, ^0^ = significantly different from the NQO or H_2_O_2_.

Oxidative stress can harm human cells by different pathways such as induction of DNA damage or apoptosis and cell death. To screen the potential protective activity of kinetin against oxidative stress induced DNA damage, oxidative stress was introduced by two genotoxic compounds with different mechanisms of action, namely, NQO and H_2_O_2_ (hydrogen peroxide); DNA damage and reduced cell viability were investigated as possible consequences. Treatment of the cells with the two compounds alone for 30 min yielded a significant induction of DNA damage in comparison to the control cells while 24 hr kinetin pretreatment protected the cells from DNA damage as measured with the comet assay (Fig 3A).

With NQO as model compound a further investigation was conducted. A washing step was inserted after the 24 hr treatment with kinetin and before the addition of NQO to HL-60 cells (Fig 3B). Again, cells were protected, indicating that the effect was at least not solely due to scavenging of NQO by kinetin in the culture medium.

Next, to investigate the protective property of kinetin aginst cell death and apotosis, two different genotoxic substances were used and two different endpoints were measured, namely, 1mM NQO to induce cell death (measured by a cell viability assay) and 10 μM Na-arsenite to induce apoptosis (microscopic evaluation of nuclear morphology and Annexin V staining).

**Fig 4:**
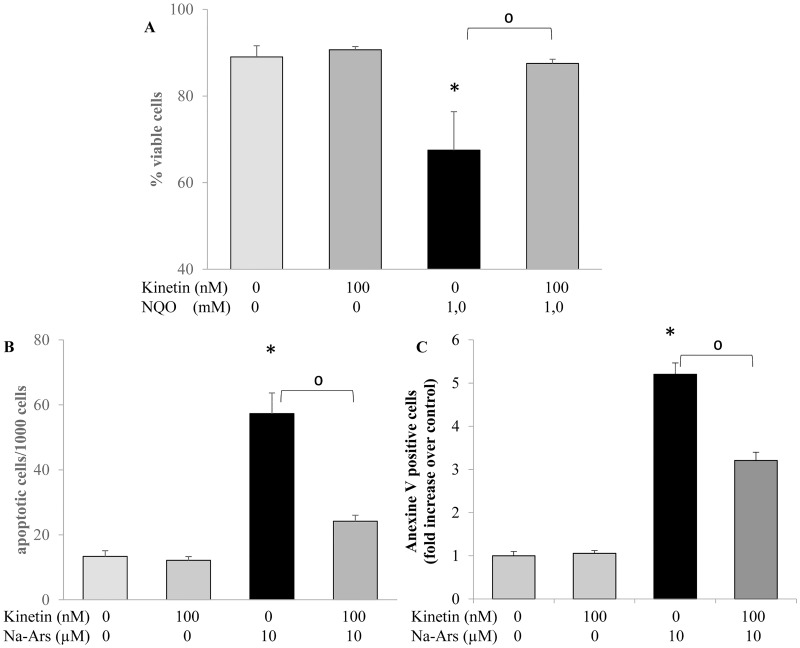
A) Anticytotoxic activity of kinetin measured by vitality test in HL-60 cells treated for 24 hr with 100 nM kinetin, and 30 min with 1.0 mM NQO and the combination of both, B) Microscopic analysis of the antiapoptotic activity of kinetin in HL-60 cells treated for 24 hr with 100 nM kinetin, 8 hr with 10 μM Na-arsenite and the combination of both. * = significantly different from control, ^0^ = significantly different from the NQO or Na-arsenite.

**Fig 5:**
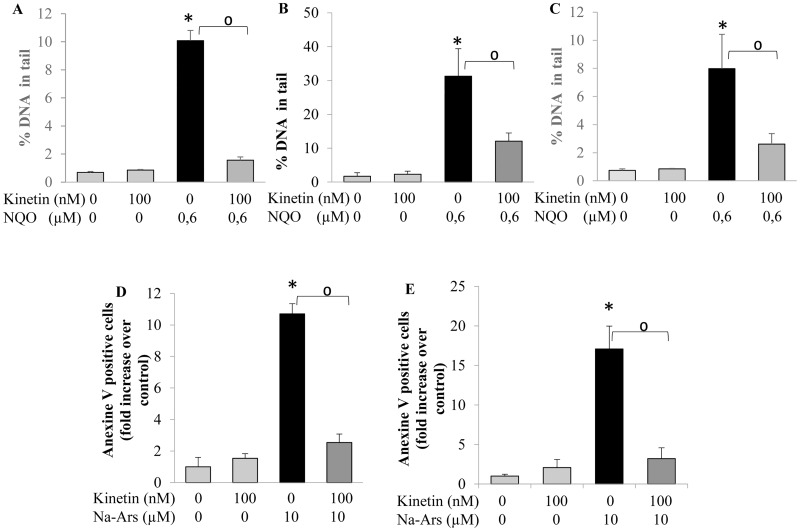
DNA damage (% DNA in tail) measured by comet assay in A) HaCaT cells, B) NRK cells, C) Isolated lymphocytes; treated for 24 hr with 100 nM kinetin, and 30 min with 0.6 μM NQO and the combination of both. Anti-apoptotic activity of kinetin in D) HaCaT cells, E) NRK cells treated for 24 hr with 100 nM kinetin, 8 hr with 10 μM Na-arsenite and the combination of both using Annexin V staining. * = significantly different from control, ^0^ = significantly different from NQO.

The treatment of the HL-60 cells with 1 mM NQO led to about 35% reduction in cell viability while upon pretreatment of the cells with kinetin, the effect of NQO was prevented and the percentage of non-viable cells was similar to the control level (Fig 4A). Cells which were treated with Na-arsenite showed an increase in apoptotic cell number in comparison to the untreated cells while pretreament of the cells with kinetin attenuated the frequency of the induced apoptosis (Fig 4B and 4C). The protective effects of kinetin against oxidative stress induced DNA damage (Fig 5A, 5B and 5C) and apoptosis (Fig 5D and 5E) was not limited to the HL60 but extended to the other normal (non-transformed) cells lines (HaCaT and NRK cells) and the isolated lymphocytes, where a similar trend of protection was observed.

Our results are in agreement with Li M et.al [29] who showed that kinetin inhibits apoptosis of aging spleen cells induced by D-galactose in rats and with Ratten et.al [30] who reported that kinetin protects against Fenton reaction-mediated oxidative damage to DNA. Verbeke et al [31] also reported the protective action of kinetin against protein oxidation and glycoxidation *in vitro*.

Several cosmetics companies formulated kinetin in preparations of skin care products and described the plant hormone CK as antiaging agent [32], without further indications to the specific mechanism. According to our results and those of others, its antioxidative capacity may provide a mechanistic basis for such an activity. Due to the absence of cytotoxicity in this dose range, the compound may also have potential for systemic applications. In fact, aiming at a treatment against familial dysautonomia, kinetin has been applied to patients at a dose of 23.5 mg/Kg/d for 28 d with promising results [9, 33]. The exogenous application of CKs and their anti-ageing effects for fibroblast cells [34–36], and the interaction between CKs and the adenosine A2A-receptor and its function as novel neuroprotectant [35] and anti-inflammatory [37] agent with immune suppressive effects have interesting immunomodulatory implications for human cells. Quite intriguingly and apart from the external application/addition of CKs to mammalian cells a recent finding on the regulation of LOG-like protein (CK-activating enzyme) by the animal pathogen *M*. *tuberculosis* [3] underscores the biological significance of CKs in animal/mammalian models of host-pathogen interactions [4]. As LOG-domain containing proteins are present in various pathogenic and non-pathogenic prokaryotic lineages [4] and thus points to the potential implications of CKs for infection by various important animal pathogens. Other applications may follow. Based on our data, we recommend an affinity based targeted small-molecule (CK) protein interaction screen together with structural modelling and molecular dynamic simulations to systematically reveal CK-binding proteins (CBPs) in mammalian cells. All these data will elucidate the responsible mechanistic pathways, and confirm further the generality of CK signalling using different cell lines and CK types.

## Abbreviations

DHE: dihydroethidium
DABCO: diazabicyclo-octan
FRAP: ferric reducing ability of plasma
GSH: glutathione
GSSG: glutathione disulfide
NQO: 4-Nitroquinoline 1-oxide
Na-Ars: sodium Arsenite
ROS: reactive oxygen species
